# Long-lasting rescue of schizophrenia-relevant cognitive impairments via risperidone-loaded microPlates

**DOI:** 10.1007/s13346-021-01099-x

**Published:** 2022-01-01

**Authors:** Elena Bellotti, Gabriella Contarini, Federica Geraci, Sebastiano Alfio Torrisi, Cateno Piazza, Filippo Drago, Gian Marco Leggio, Francesco Papaleo, Paolo Decuzzi

**Affiliations:** 1 Laboratory of Nanotechnology for Precision Medicine, Istituto Italiano Di Tecnologia, Via Morego 30, 16163 Genova, Italy; 2 Department of Biomedical and Technological Sciences, Università Di Catania, Via Santa Sofia 97, 95125 Catania, Italy; 3 Analytical Department, Consortium Unifarm, Università Di Catania, Viale A. Doria 21, 95125 Catania, Italy; 4 Genetics of Cognition Laboratory, Neuroscience area, Istituto Italiano Di Tecnologia, Via Morego 30, 16163 Genova, Italy

**Keywords:** Long-acting formulations, Microparticles, Risperidone, Schizophrenia, Drug delivery

## Abstract

Schizophrenia is a disorder characterized by cognitive impairment and psychotic symptoms that fluctuate over time and can only be mitigated with the chronic administration of antipsychotics. Here, we propose biodegradable microPlates made of PLGA for the sustained release of risperidone over several weeks. Two microPlate configurations – short: 20 × 20 × 10 μm; tall: 20 × 20 × 20 μm – are engineered and compared to conventional ~ 10 μm PLGA microspheres in terms of risperidone loading and release. Tall microPlates realize the slowest release documenting a 35% risperidone delivery at 100 days with a residual rate of 30 ng/ml. Short microPlates and microspheres present similar release profiles with over 50% of the loaded risperidone delivered within the first 40 days. Then, the therapeutic efficacy of one single intraperitoneal injection of risperidone microPlates is compared to the daily administration of free risperidone in heterozygous knockout mice for dysbindin-1, a clinically relevant mouse model of cognitive and psychiatric liability. In temporal order object recognition tasks, mice treated with risperidone microPlates outperform those receiving free risperidone up to 2, 4, 8, and 12 weeks of observation. This suggests that the sustained release of antipsychotics from one-time microPlate deposition can rescue cognitive impairment in dysbindin mice for up to several weeks. Overall, these results demonstrate that risperidone-loaded microPlates are a promising platform for improving cognitive symptoms associated to schizophrenia. Moreover, the long-term efficacy with one single administration could be of clinical relevance in terms of patient’s compliance and adherence to the treatment regimen.

**Graphical abstract:**

Single injection of long-acting risperidone-loaded µPL ameliorates the dysbindin-induced deficit in a clinically relevant mouse model of cognitive and psychiatric liability for up to 12 weeks

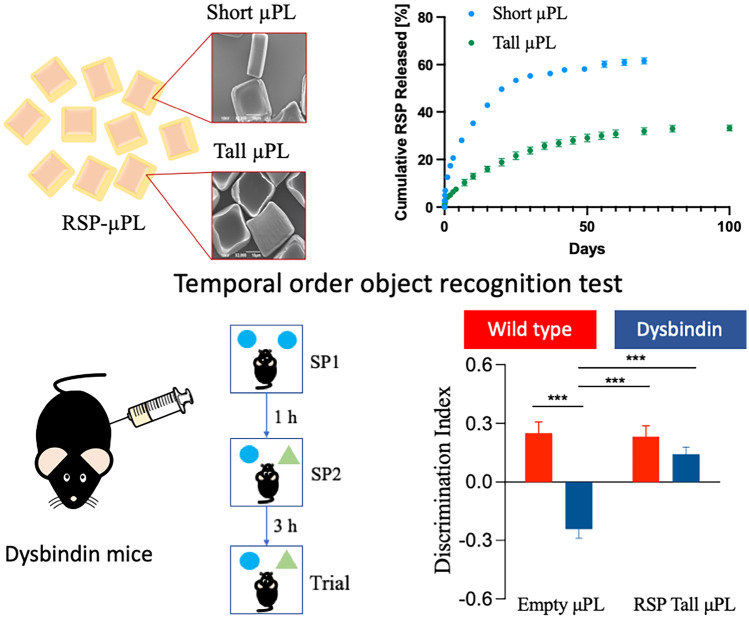

**Supplementary information:**

The online version contains supplementary material available at 10.1007/s13346-021-01099-x.

## Introduction

Schizophrenia is a chronic and debilitating neuropsychiatric disorder affecting almost 1% of the population worldwide and representing a significant health care burden with social and economic implications [1–3]. The annual costs associated with schizophrenia are elevated and connected to life-long chronic debilitations [2–4]. At present, there is no cure for schizophrenia, and current guidelines recommend life-long treatment with antipsychotic drugs in combination with psychological intervention to improve symptoms and prevent psychosis relapse [5–7]. However, due to the nature of the chronic treatments and patients phenotypes, a major limitation in the cure of schizophrenia is the non-adherence to the treatment regimen, which leads to an elevated risk of relapse, re-hospitalization, and in some cases suicide [5–7]. In addition, it is well-known that the long-term use of antipsychotics can lead to metabolic side effects as well as to the high risk of extrapyramidal side effects [8, 9].

One method to increase patient’s adherence and compliance and reduce side effects relies on the use of long-acting injectable (LAI) formulations, which have shown huge improvements in preventing relapse, hospitalization, and mortality in schizophrenic patients [6]. Importantly, LAI formulations avoid plasma drug fluctuations, thus preventing side effects due to high plasma drug levels or no effects due to low plasma levels [9, 10]. A number of LAIs already marketed or in clinical trials are meant for the long-term release of risperidone (RSP), one of the currently most largely used antipsychotic drug, and one of the few to be suggested also during pediatric and adolescent age [11, 12]. RSP has been demonstrated to be effective in improving cognitive symptoms, which are key in the pathophysiology of schizophrenia [13, 14]. In this context, the first long-acting RSP (Risperdal Consta™) was approved by FDA in 2004 [15]. This is an aqueous suspension of poly(lactic-co-glycolic acid) (PLGA) microspheres to be injected deep intramuscularly and showing a size of about 100 µm [16–18]. The RSP release profile from these large microspheres shows an initial release of ~ 2%, followed by a lag phase of approximately 3 weeks (lag phase), and finally by a continuous release lasting for about 30 days [17, 18]. In 2018, FDA approved another RSP-based LAI formulation, named the RBP-7000 (Perseris™), that would require only one subcutaneous injection every month [6, 15, 19].

Although LAIs represent a great improvement over the daily RSP administration, the aforementioned clinically approved formulations present various limitations. These include the initial lag phase in drug release requiring RSP oral supplementation; the fast release rates forcing for at least monthly injections; and the large particle size causing the use of large needles for intratissue injection [6]. Accordingly, in this work, a long-term drug delivery system based on PLGA microPlates (μPL) is designed and tested for the controlled and continuous release of RSP over a period of 3 months, following one single intraperitoneal administration. These micron-sized particles are fabricated using a top-down approach and exhibit a square base of 20 × 20 μm. The μPL height can be precisely tailored, between 10 and 20 μm, to modulate the release profile. μPL are characterized for their morphological, physico-chemical, and pharmacological features. To test their in vivo efficacy, μPL are intraperitoneally injected in heterozygous knockout mice for dysbindin-1 (Dys ±), a clinically relevant mouse model of cognitive and psychiatric liability, with proved evidence of modulatory responses to antipsychotic drugs in mice and human patients [20–24].

## Materials and methods

### **Materials**

Polydimethylsiloxane (PDMS, Sylgard 184) and elastomer were purchased from Dow Corning (Midland, MI, USA). Poly(vinyl alcohol) (PVA, Mw 31–50 kDa), poly(D,L-lactide-co-glycolide) (PLGA, lactide:glycolide 50:50, Mw 38–54 kDa), risperidone (RSP, Mw 410.40 Da), acetonitrile (ACN), and chloroform (CHCl_3_) were purchased from Sigma Aldrich (Saint Louis, MO, USA). Phosphate buffered saline (PBS) was purchased from Thermo Fisher Scientific (USA). All reagents and solvents were used as received.

### Fabrication of the µPL

**Fig. 1:**
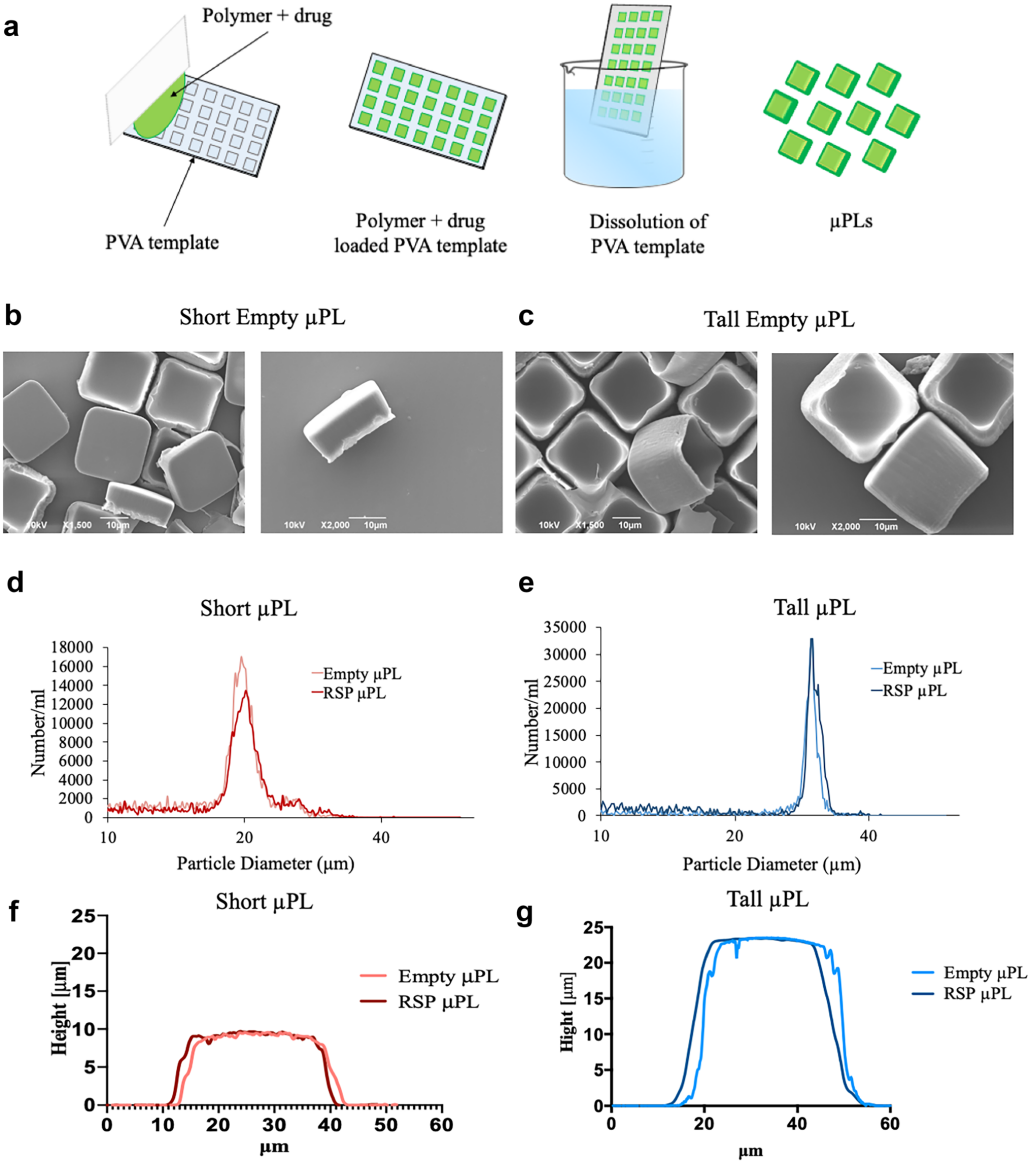
μPL fabrication process, morphological and dimensional characterization. (**a**) Sequential steps in the fabrication of 20 × 20 × 10 and 20 × 20 × 20 μm μPL. A silicon master template is fabricated via direct laser writing and replicated into a PDMS template, whose pattern is then transferred into a sacrificial PVA template. This PVA template is loaded with the polymeric paste constituting the final μPL and enclosing the payload. μPL are released and collected upon dissolution in DI water of the sacrificial PVA template (from left to right). (**b**) SEM micrographs of short and (**c**) tall μPL showing the characteristic dimensions of 20 × 20 × 10 and 20 × 20 × 20 μm. (Scale bar: 10 μm). (**d**) Size characterization of short and (**e**) tall μPL via Multisizer analyses. (**f**) Cross-section profile of short and (**g**) tall μPL acquired by optical profilometer showing the thickness of the μPL. No significant differences were highlighted between empty and RSP-μPL

RSP-loaded PLGA μPL were fabricated via a top-down approach slightly modified from previous works [25–27]. Briefly, the preparation process follows sequential replica molding steps as schematically shown in Fig. 1a. First, a master template was created via direct laser writing. The master template consists in a silicon substrate having arrays of square wells with an edge length of 20 μm and a depth of either 10 μm or 20 μm, used to obtain short and tall μPL, respectively. Each array is separated by a gap of 3 μm. Subsequently, the silicon template was replicated by covering it with a mixture of PDMS and curing agent that was allowed to polymerize for 8 h at 60 °C. Then, the PDMS template was peeled off the silicon master, and a PVA solution (5% w/v in deionized (DI) water) was deposited on the PDMS template. After water evaporation, the PVA template presented the same arrays of wells as the original silicon master mold. Finally, the wells on the PVA template were loaded with a solution of PLGA (20 or 60 mg in ACN for the short and tall μPL, respectively) and risperidone (250 μg in CHCl_3_). After solvent evaporation, the PVA template was dissolved in water, and the solution was filtered using polycarbonate filters (40 μm pore size). The final μPL were collected through sequential centrifugation steps (5000 rpm × 5 min) and stored at 4 °C until further use. Empty μPL were fabricated following the same approach, with the only difference that the PVA template was loaded with a solution containing PLGA only.

### Morphological and dimensional characterization of the µPL

The morphology and dimensions of μPL were characterized using different techniques. Specifically, μPL shape and size analysis were carried out via scanning electron microcopy (SEM, JEOL JSM-6490LA). Briefly, a drop of sample was deposited on a silicon support and uniformly sputtered with gold to increase the contrast and reduce sample damaging. An acceleration voltage of 10 keV was used for SEM image acquisition. Additionally, μPL average size and distribution were obtained via volume impedance measurements on a Multisizer 4 COULTER particle counter (Beckman Coulter, CA). μPL were resuspended in an electrolyte solution and analyzed using a 100 μm capillary. Furthermore, topographical analysis was carried out to assess the average thickness of the particles using an optical profilometer.

### Degradation of the µPL

The degradation of μPL over time was evaluated in vitro via SEM and Multisizer analysis. Briefly, μPL were incubated in PBS under mild mechanical stirring at 37 °C. At different time points, samples were analyzed to asses morphological and structural changes [27].

### Biopharmaceutical characterization of the µPL

The RSP loading and encapsulation efficiency (LE and EE, respectively) into RSP-μPL were evaluated by dissolving the μPL in ACN/H_2_O (1:1 v/v) and analyzing the solution via high performance liquid chromatography (HPLC) (Agilent 1260 Infinity, Germany), equipped with a 100 μl loop. The mobile phase consisted in ACN + 0.1% TFA (v/v) and DI water + 0.1% TFA (v/v) at a ratio of 57/43 v/v and pumped at an isocratic flow rate of 0.3 ml/min. The analysis was performed by using a C18 column (2.1 × 10 mm, 3.5 μm particle size, Agilent Eclipse Plus, USA) at a detection wavelength of 280 nm. Two different standard calibration curves were created starting from standard aliquots of RSP in either ACN/DI water or PBS over the range of 0.6 to 10 µg/ml. In addition, the limit of detection (LoD) and the limit of quantification (LoQ) of the HPLC method used to quantify RSP were evaluated starting from the standard calibration curves. Specifically, data of the standard curves (concentration – µg/ml, and signal – area) were analyzed by a linear regression algorithm, and LoD and LoQ evaluated based on the standard deviation of the response and the slope, according to the following equations [28]:

1 $$LoD=\frac{3.3\sigma }{S}$$

2 $$LoQ=\frac{10\sigma }{S}$$

where $\sigma$ and S are the standard deviation of the response and the slope of the calibration curve, respectively. Values of LoD and LoQ are shown in supplementary Table S1.

The amount of RSP loaded and encapsulated into the μPL was calculated interpolating a standard calibration curve. The loading (%LE) and encapsulation efficiency (%EE) were quantified using the following equations [27, 29]:

3 $$\mathrm{LE}\left(\%\right)=\frac{\mathrm{RSP}\;\mathrm{weight}\;\mathrm{in}\;\mathrm{particles}}{\mathrm{Total}\;\mathrm{weight}\;\mathrm{of}\;\mathrm{particles}}\times100$$

4 $$\mathrm{EE}\left(\%\right)=\frac{\mathrm{RSP}\;\mathrm{weight}\;\mathrm{in}\;\mathrm{particles}}{\mathrm{RSP}\;\mathrm{initial}\;\mathrm{feeding}\;\mathrm{amount}}\times100$$

To study RSP release, short and tall μPL were incubated in 500 μl PBS under mechanical stirring at 37 °C. At predetermined time points, the μPL were centrifuged at 500 RPM, the supernatant removed, and 500 μl of fresh PBS added. To quantify the amount of RSP released over time, the supernatant was analyzed via HPLC using the same method described above. To interpret the RSP release profile, the experimental data were fitted to the two-phase Weibull model equation [30]:

5 $$\frac{({\mathrm{M}}_{\mathrm{t}})}{({\mathrm{M}}_{\infty })}=1-\mathrm{exp}(-\mathrm{a}\times {\mathrm{t}}^{\mathrm{b}})$$

where *M*_*t*_ and *M*_*∞*_ represent the amount of drug released at time *t* and infinite, respectively, *a* is a constant based on the system, and the exponent *b* is an indicator of the mechanism of transport through the polymer matrix. Values of *b* ≤ 0.75 indicate Fickian diffusion, while a combined mechanism is related to b values in the range of 0.75 < *b* < 1. For values of *b* > 1, the drug transport follows a complex release mechanism [30].

### In vivo* studies*

#### Animals

All procedures were approved by the Italian Ministry of Health (permits n. 230/2009-B and 107/2015-PR) and the local Animal Use Committee and were conducted in accordance with the Guide for the Care and Use of Laboratory Animals of the National Institute of Health and the European Community Council Directives.

Behavioral analyses were carried out on 3- to 7-month-old male dysbindin heterozygous mutant mice (Dys ±) and their wild-type littermates (Dys + / +). The breeding scheme used to obtain the experimental mice involved mating Dys ± male mice with Dys + / + females. Dys + / + mice were used as female breeders in order to avoid altered maternal behavior. Animals were group-housed (3–5 per cage) and kept at constant room temperature (22 ± 2 °C) and relative humidity (60%), with a 12 light/dark cycle (7 am–7 pm). Animals had free access to food and water. Distinct cohort of animals was used for each experiment. Mice were handled on alternate days during the week preceding the start of the treatment. All the experiments were carried out during the light phase. At least 1 h prior the beginning of the test, animals were allowed to acclimatize to the testing room.

#### Drug and treatment

RSP was dissolved in 10 μl of acetic acid, made up to volume with physiological saline (0.9% NaCl), and pH was adjusted to 6 with 0.1 M NaOH. Each mouse received an intraperitoneal (IP) injection of 10 ml/kg, corresponding to a dose of 0.1 mg/kg [20, 21]. Animals were treated with RSP or saline once daily for 14 consecutive days before the behavioral analysis.

Mice treated with RSP-μPL received a single injection of either short or tall μPL on day 0. The amount of injected μPL contained the same amount of RSP that was administered once daily for 14 days (0.1 mg/kg/14 days). μPL were resuspended in saline solution, and 150 μl of the particle suspension was IP injected. Animals were treated with either short and tall RSP-μPL or short and tall empty μPL, and behavioral analyses were carried out at predetermined time points.

#### Temporal order object recognition test

The temporal order object recognition (TOR) test was performed as previously described [20, 31–33]. Briefly, the task was carried out in an evenly illuminated (25 ± 5 lx) open-field arena (40 × 40 × 40 cm, Ugo Basile, Gemonio, Italy). To minimize stress-related behavior and neophobia, mice were habituated to the experimental apparatus for 4 days (10 min per day). The TOR test consists of two sample phases (SP1 and SP2) and a test phase. During SP1 and SP2, mice were allowed to explore two identical objects (different for each sample phase) for 5 min. During the test phase, mice were allowed to explore an identical copy of the objects presented in SP1 and SP2, respectively. All the object used were plastic Duplo blocks (LEGO©) different in shape, color, and size (ranging from 9 × 8 × 7 cm to 12 × 11 × 10 cm). The objects were placed at the corners of the arena, 8 cm from the sidewalls. To avoid olfactory cues, the objects were cleaned with 10% ethanol solution. The sample phases and the test phase were recorded using a camera (Sony videocam PJ330E). The time spent exploring each object was scored from the recorded videos, when mouse was facing the object 1 cm away. Climbing or rearing against the objects was not considered as an exploration. In addition, mice that spent less than 2 s exploring the objects were excluded from the analysis. The discrimination between the objects was evaluated using the discrimination index (DI) calculated as:

6 $$DI=\frac{Ti-Tr}{Ttot}$$

where *T*_*i*_ is the less recently presented object exploration time, *T*_*r*_ is the most recently presented object exploration time, and *T*_tot_ is the total exploration time.

If the TOR memory is intact, during the test trial, mice should spend more time exploring the less recently presented object. All the results are expressed as the mean ± standard error of the mean (SEM).

#### In vivo RSP concentration

Twelve weeks after the single injection of tall RSP-μPL, mice were sacrificed, and the whole blood collected to investigate the presence of RSP, if any. The whole blood was stored at room temperature for at least 1 h and centrifuged at 1000* g* for 15 min to collect the serum. Serum was then stored at − 80 °C. Subsequently, RSP was extracted from mouse serum by acetonitrile protein precipitation method. Serum samples were extracted using 100 µl of acetonitrile. The extracts were then centrifuged for 15 min at 6.000 rpm at 4 °C. The supernatant was transferred in a glass vial and then injected into LC–MS/MS for analysis. Sample analysis was carried out using an Agilent 6410-A series triple quadrupole mass spectrometry with an electrospray ionization source (Agilent Technologies, Santa Clara, CA, USA). The analysis was performed using a Phenomenex Kinetex C8 100 Å analytical column (50 × 2.1 mm, 2.6 µm). The mobile phase consisted in DI water + 0.5% acetic acid (v/v) and acetonitrile at a ratio of 50/50 v/v, and pumped at a flow rate of 0.2 ml/min. RSP was analyzed in positive ionization mode following multiple reaction monitoring (MRM) transitions: 411.3 → 191.3 m/z. The amount of RSP in serum was calculated interpolating a calibration curve in the concentration range of 0.5–30.87 ng/ml.

### Statistical analysis

One-way ANOVA (analysis of variance) was performed to analyze the difference among the three particle configurations in terms of loading, encapsulation efficiency, and RSP released. One- or two-way ANOVA was performed to analyze the effects of the treatments in the cognitive performance during the TOR test. Newman-Keuls or Bonferroni post-hoc test was used for comparison among groups when the overall ANOVA showed statistically significant differences. The treatment effect induced by empty or RSP-μPL in Dys ± mice at 4-weeks post-injection was also analyzed by Student *t* test. The level of significance was set at *p* < 0.05. All the data were analyzed using GraphPad Prism 8.0.2 (GraphPad Software, La Jolla, CA, USA).

## Results

### Fabrication and morphological characterization of the μPL

Two configurations of RSP-loaded μPL – short μPL and tall μPL – were fabricated following a multi-step, top-down approach and previously described by the authors [25, 26]. In brief, μPL are realized using a template-based fabrication strategy involving a silicon master template, whose wells’ geometry dictate the particle morphology, replicated in an intermediate PDMS template and eventually into a sacrificial PVA template (Fig. 1a). The wells in the PVA template are accurately filled with a polymeric paste (PLGA) dissolved in organic solvent (acetonitrile) carrying the therapeutic molecule (RSP). The PVA template is dissolved in water releasing the RSP-μPL in solution.

The two μPL configurations were characterized via scanning electron microscopy (SEM), Multisizer Coulter counter, and profilometric analyses for their morphological properties. The SEM images of Fig. 1b, c show well-defined short and tall μPL with a common edge size length of 20 μm and a height of 10 and 20 μm, respectively. The different μPL heights can be readily appreciated in the lateral magnified pics of the microparticles laying on their sides. No porous structure was observed on the particle surface at this magnification level. Moreover, as expected, no significant difference in morphology was documented when comparing empty μPL and RSP-μPL (Fig. S1a, b). The Multisizer analysis showed single peaks around 20 μm and 30 μm for the short and tall μPL, respectively, with again no appreciable difference between empty and RSP-μPL (Fig. 1d, e). Additional topographical analyses carried out with an optical profilometer confirmed the μPL geometrical features as documented by the cross-section profiles of Fig. 1f, g and in the false-coloring 3D reconstruction in Fig. S1c − f, for both the short and tall μPL. Indeed, one major advantage of the template-based fabrication strategy is the accurate control in particle morphology, which cannot be generally achieved with bottom-up fabrication strategies.

### Degradation of the µPL

**Fig. 2:**
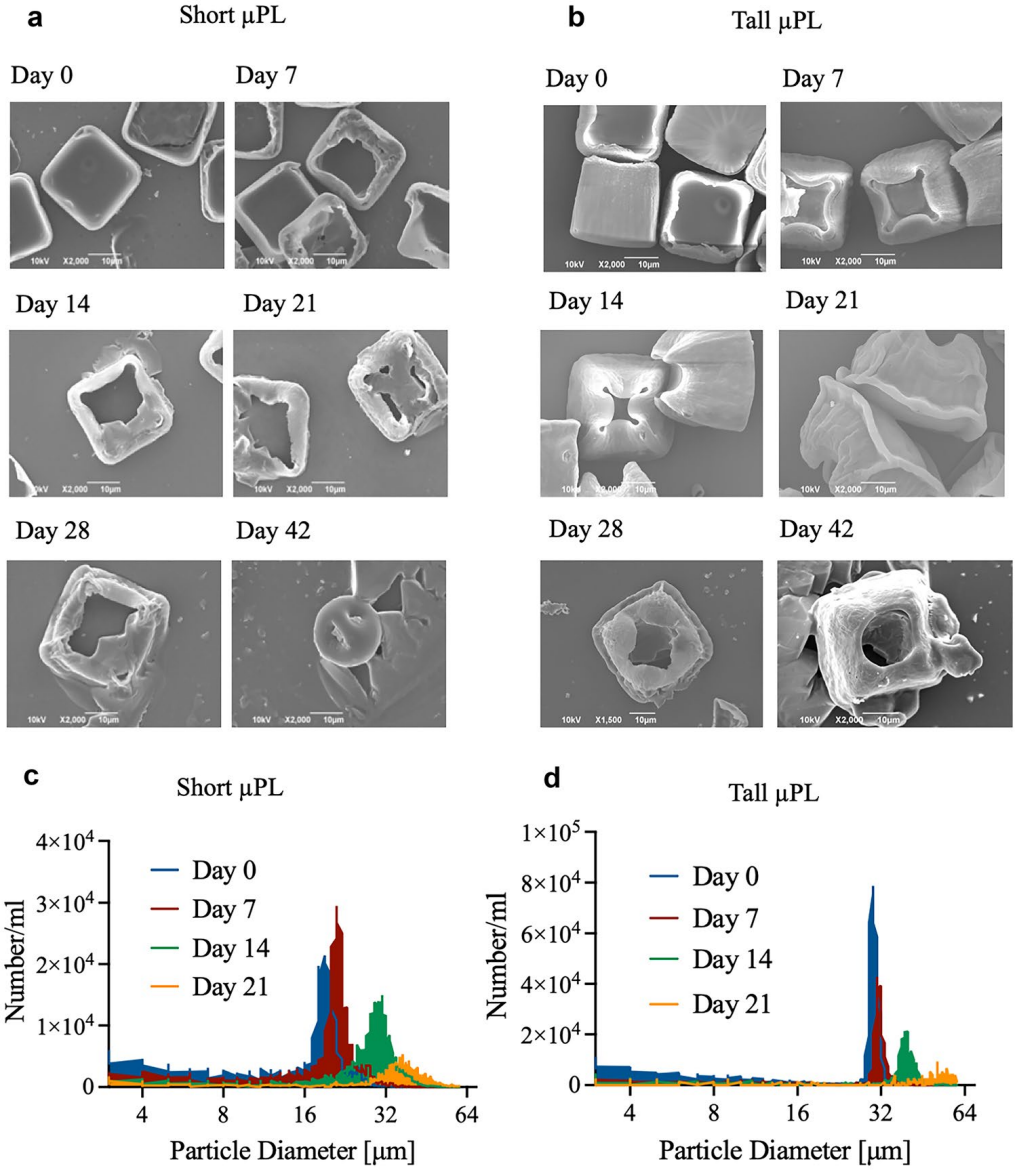
Degradation of μPL under physiological conditions. (**a**) SEM images representing the degradation of short and (**b**) tall μPL over time. (**c**) Degradation of short and (**d**) tall μPL analyzed via the Multisizer

To investigate the biodegradation of the PLGA polymer matrix, both short and tall μPL were incubated in PBS at 37 °C, and their morphology was assessed over time to document any possible alterations. Figure 2a, b show SEM images of the μPL acquired at different time points, from 0 up to 42 days, post-incubation.

On day 0, both short and tall μPL appeared as well-defined, squared particles as from Fig. 1b − g. On day 7, however, both short and tall μPL started to present some preliminary sign of biodegradation as manifested by the rounding of the originally sharp corners and the progressive transition from a square base to an overall rounded shape. At longer time points, the process started to affect also the core of the short μPL with a degeneration of their structure into a round microparticle already on day 42. The tall μPL followed overall the same behavior but at lower degradation rates, likely due to the larger amount of PLGA used to realize the tall versus short μPL. Nonetheless, even for the tall μPL, the microparticles tended to progressively lose their original squared shape to become more globular towards the end of the observation period (Fig. S2). This behavior was quantified via a Multisizer Coulter counter analysis returning the overall size spectrum distribution for the short and tall μPL over time (Fig. 2c, d). This shows a progressive shift of the characteristic peak towards a larger average size and a reduction of the peak value and overall area under the curve. The shift of the peak has to be associated with the change in microparticle structure and possible coalescence with other particles or debris. The reduction in peak value is directly correlated with the number of particles. Multisizer data are referred to the degradation of particles from day 0 to day 21. Note that longer time point measurements were not possible due to the change in μPL morphology that made the sample unsuitable for the analysis.

### Biopharmaceutical characterization of the μPL

Short and tall μPL loaded with RSP were characterized in terms of RSP loading (LE%), defined as the ratio between the loaded mass of RSP and the total particle mass; encapsulation efficiency (EE%), defined as the ratio between the loaded mass of RSP and the initial RSP input; as well as for their ability to control the release of RSP over time. A direct comparison with PLGA microspheres was also performed. Specifically, ~ 10 μm PLGA spherical particles (μS) were realized using a standard homogenization protocol [34, 35]. Figure S3 shows electron microscopy images, DLS, and Multisizer analysis for the ~ 10 µm microspheres.

**Fig. 3:**
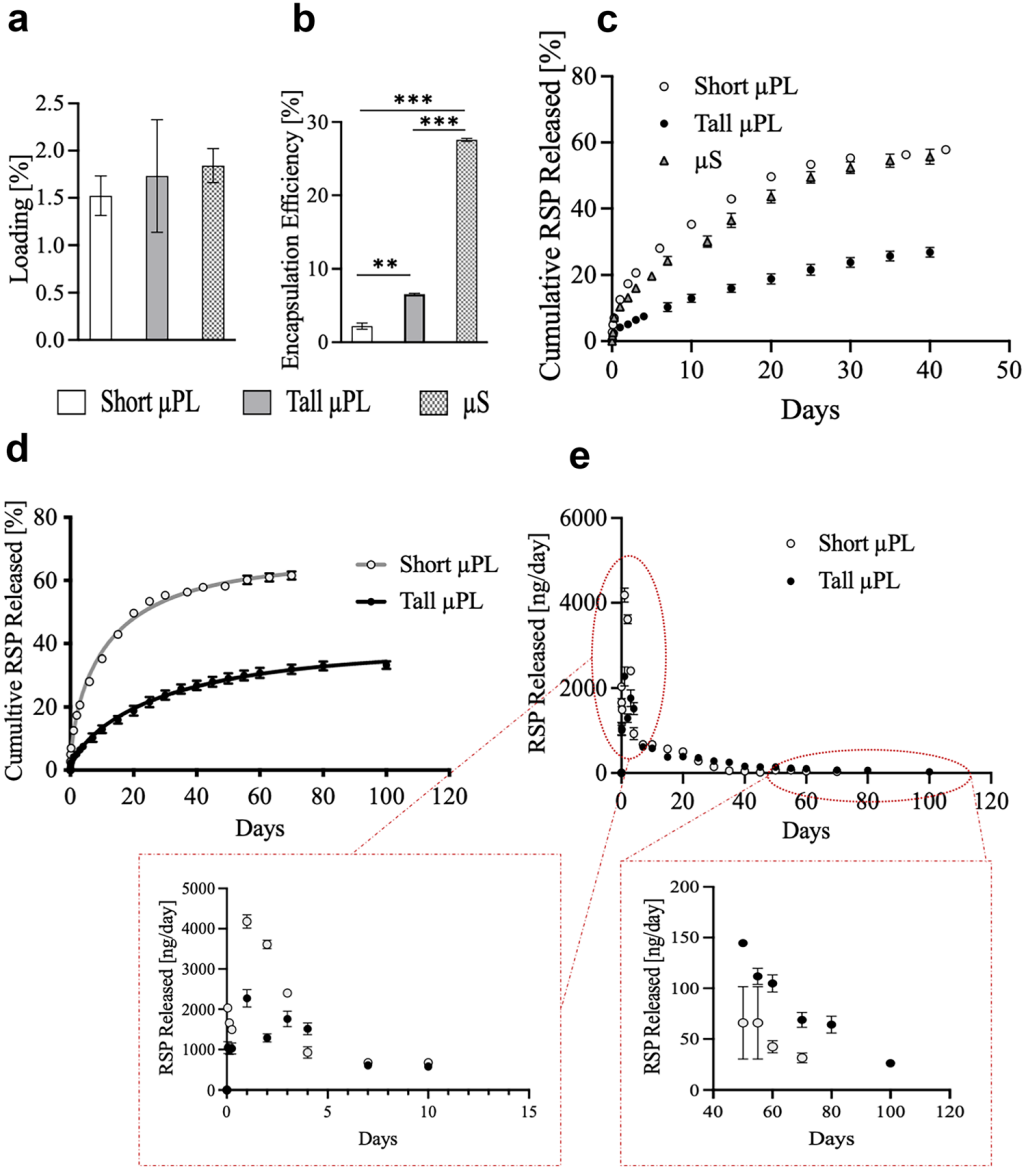
Pharmacological characterization of μPL. (**a**) RSP loading into short µPL, tall μPL, and µS. (**b**) RSP encapsulation into short µPL, tall μPL, and µSpheres. Statistical significance was determined by one-way ANOVA: **p < 0.01 tall µPL vs short µPL, ***p < 0.001 µS vs sort µPL and µS vs tall µPL. (**c**) 40-day cumulative release profiles of RSP from short µPL, tall µPL, and µS (37 °C in PBS). (**d**) Cumulative and (**e**) daily release profiles of RSP from short and tall μPL (37 °C in PBS). Green and black lines in (**d**) represent the Weibull function fitting; 95% confident band. Data are expressed as the mean ± standard deviation (*n* = 3). Statistical significance was determined by one-way ANOVA: *p < 0.05, ***p < 0.001, §*p* < 0.0001, #*p* < 0.00001. Details of the statistical analysis are reported in Table S2

No statistically significant differences were observed in terms of loading among the three different microparticles, with 1.52 ± 0.20% for the short RSP-μPL; 1.73 ± 0.59% for the tall RSP-μPL; and 1.84 ± 0.18% for the μS, respectively (Fig. 3a – one-way ANOVA: *p* = 0.685 short µPL vs tall µPL, *p* = 0.837 short µPL vs µS, *p* = 0.799 tall µPL vs µS). On the other hand, significant differences were documented for the encapsulation efficiency. The drug encapsulated within the RSP-μS (27.57 ± 0.20%) was significantly higher than for the tall RSP-μPL (6.55 ± 0.13%) and the short RSP-μPL (2.21 ± 0.43%), respectively (Fig. 3b – one-way ANOVA, ***p < 0.001). Moreover, the encapsulation efficiency of the tall µPL was significantly higher than the encapsulation efficiency of the small µPL (Fig. 3b – one-way ANOVA: **p < 0.01).

The RSP release kinetics was determined under physiological conditions (PBS, pH 7.4, 37 °C) in a 500 μl volume to better mimic the confined space for intratissue deposition. The release profiles for the two μPL configurations and the μS are shown in Fig. 3c in terms of percentage of drug released over the first 40 days of observation. Tall RSP-μPL showed the slowest release profile with less than 30% (26.85 ± 1.50%) of the loaded drug being released within the first 5 weeks of observation. In the same time period, short RSP-μPL and RSP-μS released over 50% (57.81 ± 0.85% and 55.69 ± 2.28%, respectively) of the loaded drug (one-way ANOVA: #*p* < 0.00001 short µPL vs tall µPL, §*p* < 0.0001 µS vs tall µPL). Given the similarity between the release profiles for the short RSP-μPL and RSP-μS (*p* = 0.21 – no significant differences), more details are acquired for the short and tall RSP-μPL in terms of cumulative and instantaneous drug released, as shown in Fig. 3d, e, respectively. Within the first 24 h of incubation, about 12% of RSP was released from the short μPL as opposed to 4% for the tall μPL (one-way ANOVA: ***p < 0.001), while at 50 days, about 60% and 29% of RSP was released from the short and tall μPL, respectively (one-way ANOVA: #*p* < 0.00001). For the tall μPL, the drug molecules were continuously delivered over time to reach just about 33% of release at 100 days (Fig. 3d). The daily release of RSP from short and tall μPL is shown in Fig. 3e. For the short RSP-μPL, the drug is rapidly released within the first week reaching a peak of 4000 ng/ml at day 1 that drops rapidly to 1000 ng/ml at day 5 and continuously decreases down to about 50 ng/ml around day 60. A similar trend is documented for the tall RSP-μPL but with a more moderate variation in daily release rate. Specifically, a peak of only 2000 ng/ml is detected at day 1 followed by a drop to 1000 ng/ml at day 7 that stays quasi-constant till day 25. The daily release rate continuously decreases till 50 ng/ml at day 100.

The release kinetics data were interpolated using the Weibull equation to extrapolate the RSP release mechanisms for both short and tall μPL. The Weibull model parameters (a, b) and the corresponding fitting accuracy *R*^2^ for the in vitro release profiles are reported in Table 1. In this work, the fitting was successful for the entire set of data, with a *R*^2^ of 0.9960 and 0.9979 for short and tall µPL, respectively. The values of *b* obtained for the best fit were 0.6557and 0.6766 for short and tall µPL, respectively. These values suggest that µPL provide a sustained release of RSP based on a diffusion-controlled mechanism through the PLGA matrix.

**Table 1:** Weibull model parameters (a, b) and fitting accuracy *R*^2^ for RSP release from short and tall µPL

µPL	*a*	*b*	*R*^2^
Short RSP µPL	0.201	0.655	0.99
Tall RSP µPL	0.112	0.677	0.99

Weibull model equation: $\frac{({M}_{t})}{({M}_{\infty })}=1-exp(-a\times {t}^{b})$

This implies that drug release from the μPL into the surrounding aqueous environment would depend on the molecular weight of the drug as well as on the size of the pores in the PLGA matrix rather than its biodegradation. These results are also in line with the release mechanism of other small molecules from similar microparticles [27]. In comparing the short and tall μPL, one can observe a doubling in volume (μPL height increasing from 10 to 20 μm) and a three-time increase in PLGA mass (from 20 to 60 mg) used during the fabrication process. Therefore, it can be argued that the slower release kinetics associated with the tall μPL should be associated with their more compact structure.

### Temporal order object recognition (TOR) test

#### Single injection of short RSP-μPL

**Fig. 4:**
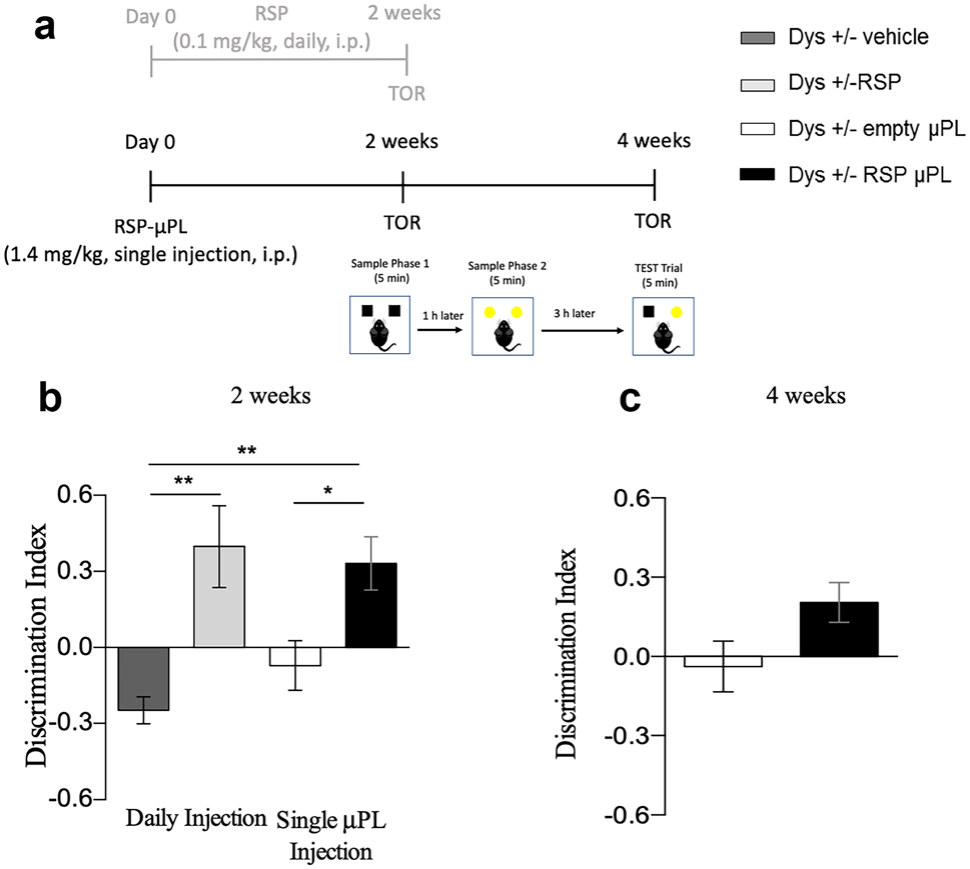
Single injection of short RSP-μPL improves cognitive abilities of Dys ± in mice in the TOR task. Each experimental group received free vehicle (*n* = 7, dark gray bars) or free risperidone (*n* = 7, light grey bars) for 14 days, and short empty (*n* = 6, white bars) or RSP-μPL (*n* = 6, black bars) only once on day 0. (**a**) Experimental timeline. (**b**) Discrimination index showed by Dys ± mice during the 5-min test phase 2 weeks post-treatment. (**c**) Discrimination index showed by Dys ± receiving either empty or RSP-μPL during the 5-min test phase 4 weeks post treatment. The values are the means ± s.e.m. One-way ANOVA: treatment effect *p < 0.05, **p < 0.005

In order to investigate if a single injection of RSP-μPL could ameliorate dysbindin-induced deficit, Dys ± mice received either free RSP (or vehicle only) or RSP-μPL (or empty μPL). The free drug or vehicle were administered intraperitoneally (0.1 mg/kg) on a daily basis for 2 weeks, whereas the RSP-μPL or empty μPL were deposited i.p. once at the beginning of the experiment for a total equivalent RSP dose of 1.4 mg/kg, to match the total amount of free drug received in 2 weeks. TOR tests are conducted at 2- and 4-week post-treatment initiation, as described in Fig. 4a.

At all the time points, Dys ± mice treated with short empty μPL were not able to discriminate between the two objects presented at different time, confirming previously found recency memory cognitive impairment (Fig. 4b, c). This suggests that the short μPL per se had no effect. At 2 weeks, the treatment with both free RSP and RSP-μPL did not affect the total time (*T*_*tot*_) mice spent to explore the two objects (F_(3,22)_ = 1.276, *p* = 0.3070) as shown in Fig. S4a, but it is effective in selectively improving TOR cognitive performances (F_(3,22)_ = 7.943, *p* = 0.0009). Specifically, at 2 weeks, Dys ± mice treated with daily injections of free RSP showed a significantly higher DI when compared to the vehicle-treated Dys ± mice (**p < 0.01) (light vs dark gray bars – Fig. 4b). Notably, also Dys ± mice treated with a single injection of short RSP-μPL improved their TOR performances, showing an increased DI that was significantly higher (*p < 0.05) than that of the Dys ± mice receiving a single injection of empty μPL (black vs white bars – Fig. 4b). Importantly, there were no significant differences between the TOR performance of Dys ± mice treated with daily injections of free RSP and Dys ± mice treated with a single injection of short RSP-μPL (black vs light gray bars – Fig. 4b). This result suggests that the μPL-based system helped ameliorate the dysbindin-induced cognitive deficits similarly to the free drug, with one single injection of particles releasing a lower amount of drug than the conventional dose. TOR was also performed at 4-week post-treatment initiation. Similar to the 2-week post-treatment, there was no significant difference in the time spent by the mice to explore the two objects (*p* = 0.5358) (Fig. S4b). However, even if Dys ± mice treated with short RSP-μPL showed improvement in the TOR performances over Dys ± mice treated with short empty μPL (Fig. 4c), the difference between the two groups was not significant (*p* = 0.6099).

#### Single injection of tall RSP-μPL

**Fig. 5:**
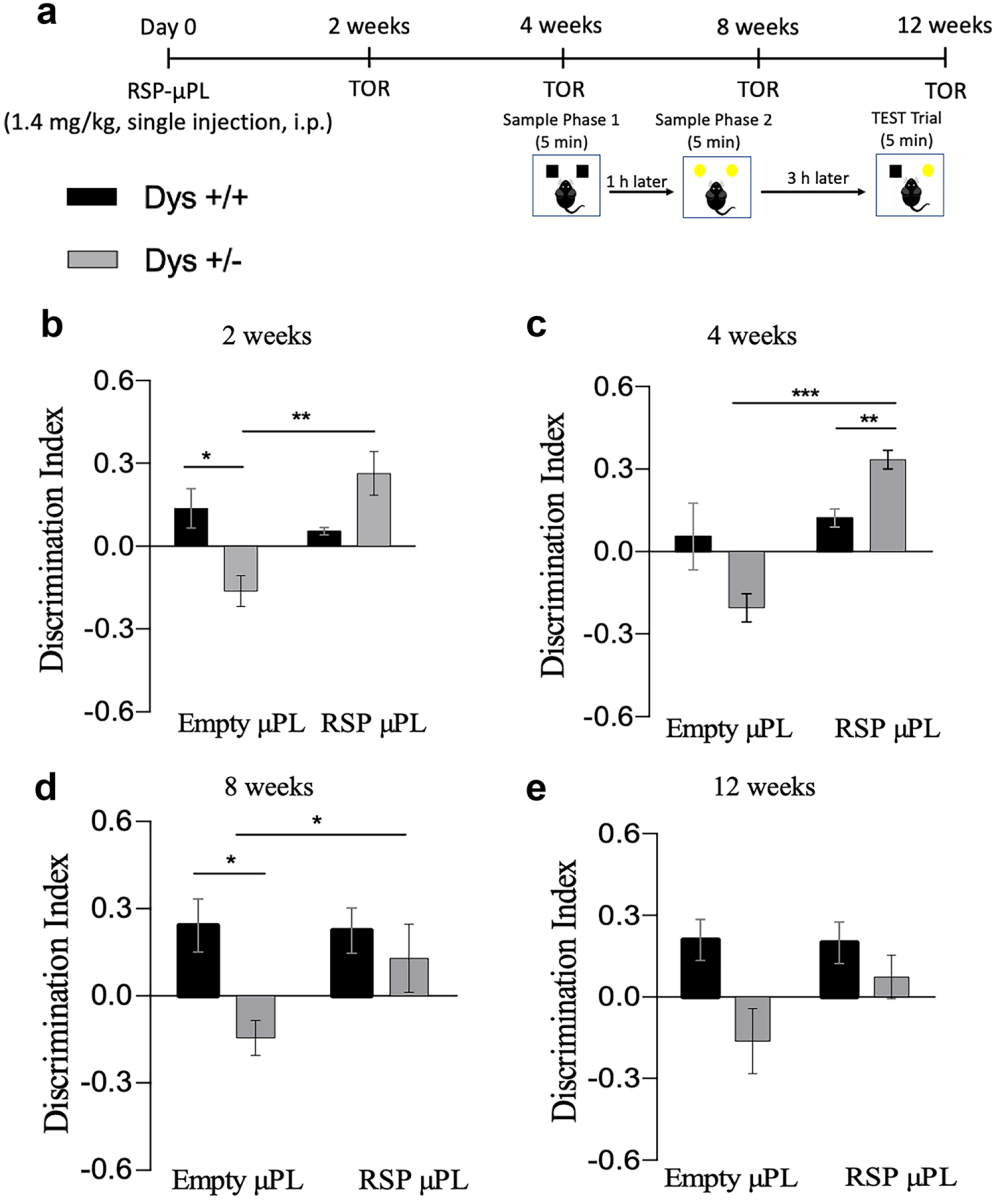
Single injection of tall μPL improves the cognitive abilities of Dys ± mice in the TOR task. Each experimental group (*n* = 6) received either empty or RSP-μPL only once on day 0 and were tested in the TOR task at 2-, 4-, 8-, and 12-week post-injection. (**a**) Experimental timeline. (**b**) Discrimination index showed by mice during the 5-min test phase at 2, (**c**) 4, (**d**) 8, and (**e**) 12 weeks, respectively. The values are the means ± s.e.m. Two-way ANOVA: treatment effect *p < 0.05, **p < 0.01, ***p < 0.001. At 2- and 4-week post-injection, the discrimination performance was affected by the treatment (2 weeks – treatment effect: F_(1, 13)_ = 7256, *p* = 0,0184; 4 weeks – treatment effect: F_(1, 13)_ = 20.55, *p* = 0,0006), at 8- and 12-week post-injection the discrimination performance was impacted mainly by the genotype (8 weeks – genotype effect: F_(1,13)_ = 6.506, *p* = 0.0242; 12 weeks – genotype effect: F_(1,13)_ = 7.739, *p* = 0.0156)

In these studies, Dys ± mice and their wild-type (Dys + / +) littermates are injected intraperitoneally on day 0 with tall empty μPL or tall RSP-μPL (1.4 mg/kg drug equivalent dose), according to the timeline shown in Fig. 5a.

The mice were subjected to the TOR tests at 2-, 4-, 8-, and 12-week post-treatment initiation. At all the time points, treatment with tall RSP-μPL did not lead to any difference in the total time spent by the mice to explore the two objects during the 5-min test trial (Fig. S5a − d), but it was effective in rescuing the cognitive impairment. Specifically, Dys ± mice treated with the RSP-μPL showed an increased DI when compared to Dys ± mice treated with the empty μPL (Fig. 5b − e). However, the difference between the two was statistically significant at 2 (Newman-Keuls post-hoc: **p < 0.001 vs empty μPL), 4 (Newman-Keuls post-hoc: ***p < 0.0001 vs empty μPL), and 8 weeks (Newman-Keuls post-hoc: *p < 0.05 vs empty μPL) only, and not significant at 12 weeks. Second, the therapeutic effect of the tall RSP-μPL on the Dys ± mice was compared to the effect of both the tall empty μPL and the tall RSP-μPL on the Dys + / + mice. No significant difference was observed at all four times points (Fig. 5b − e), thus implying that the RSP-μPL alone can rescue the animal behavior and that one single administration of RSP-μPL at day 0 returns Dys ± mice behaving just like the Dys + / + mice up to 12 weeks.

**Fig. 6:**
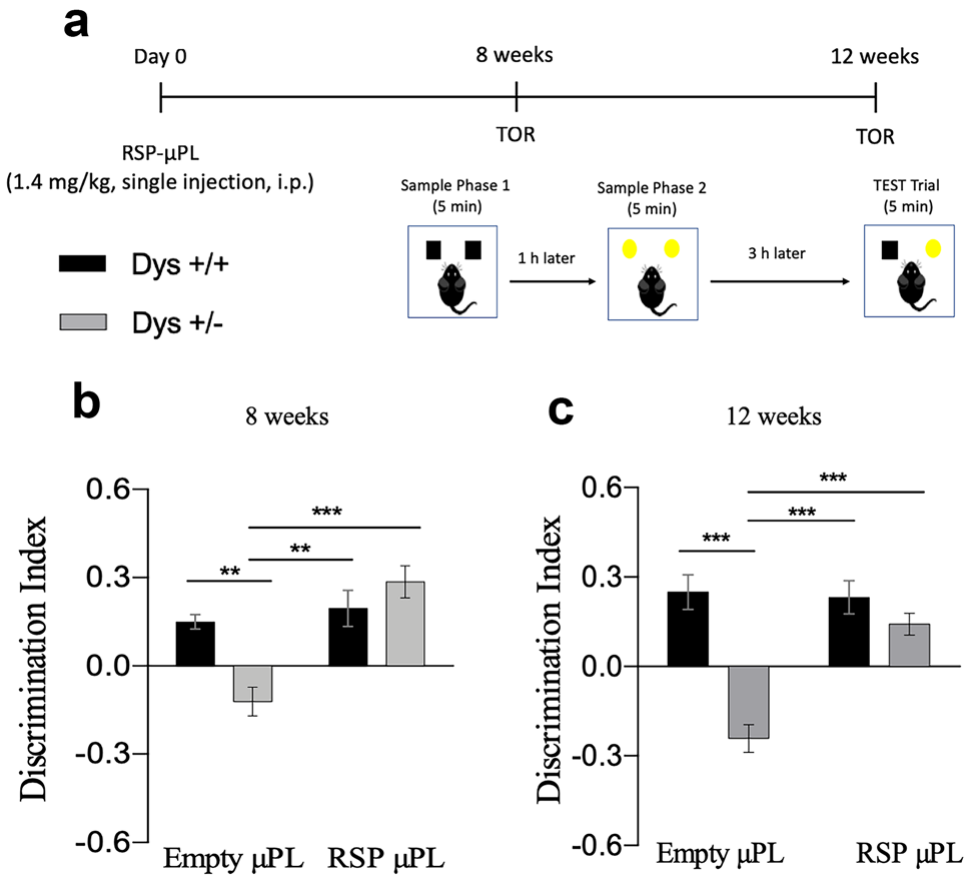
Single injection of tall μPL improves the cognitive abilities of Dys ± mice in the TOR task. Each experimental group received either empty (Dys + / + *n* = 6, Dys ± *n* = 10) or RSP-μPL (Dys + / + *n* = 6, Dys ± *n* = 10) only once on day 0 and were tested in the TOR task at 8- and 12-week post-injection. (**a**) Experimental timeline. (**b**) Discrimination index showed by mice during the 5-min test phase at 8, (**c**) 12 weeks, respectively. The values are the means ± s.e.m. Two-way ANOVA: treatment effect **p < 0.01, ***p < 0.001

To assess if the tall RSP-μPL would progressively lose their efficacy over time or if there was any kind of habituation of the animals to the TOR tasks, Dys ± mice and Dys + / + mice were injected with tall RSP-μPL or empty μPL on day 0. Then, their behavioral performances are tested only at 8- and 12-week post-injection, according to the schematic of Fig. 6a.

As previously shown, treatment with tall RSP-μPL did not lead to any difference in the total time spent by the mice to explore the two objects during the 5-min test trial (Fig. S6a, b). Importantly, a single injection of tall RSP-μPL was effective in restoring the recency memory impairment in Dys ± mice, with a DI that was significantly higher than that of Dys ± receiving tall empty μPL, at both 8 (Bonferroni post-hoc: ***p < 0.0001 vs empty μPL) and 12 weeks (Bonferroni post-hoc: ***p < 0.0001 vs empty μPL) (Fig. 6b, c). Notably, at both time points, the cognitive performance was impacted by the treatment (8 weeks – treatment effect: F_(1,28)_ = 17.76, *p* = 0.0002; 12 weeks – treatment effect: F_(1,28)_ = 16.66, *p* = 0.0003) and by the interaction between treatment and genotype (8 weeks – treatment × genotype effect: F_(1,28)_ = 11.29, *p* = 0.0023; 12 weeks – treatment × genotype effect: F_(1,28)_ = 12.90, *p* = 0.0009). These results suggest that RSP-μPL can have a prolonged effect up to 3 months upon single administration, leading to an overall improvement of the cognitive performances of mice carrying dysbindin-induced deficit.

It should be here highlighted that for all the TOR tests (Figs. 5 and 6), Dys ± mice receiving a single injection of tall empty μPL showed no discrimination, indicating the presence of a cognitive impairment and confirming that the tall μPL too per se had no effect. Notably, for all the TOR tests, the administration of tall empty µPL had no negative outcome on the cognitive performances of Dys + / + mice, reinforcing the fact that the µPL per se did not lead to any side effect.

Finally, the RSP concentration was assessed through HPLC MS/MS quantitative determination 12 weeks after a single injection of tall RSP-μPL. The analysis revealed a serum concentration of 6.40 ± 1.26 ng/ml and 4.90 ± 0.77 ng/mL in RSP-μPL treated Dys + / + and Dys ± mice, respectively (Fig. S7), which appears to be comparable to the daily RSP release measured in vitro at 100 days (Fig. 3e). Non-detectable RSP concentration was confirmed for the empty μPL treated mice.

## Discussion

The current treatments for schizophrenia rely on the life-long administration of antipsychotic drugs, such as risperidone, in combination with psychological intervention. Indeed, given this premises, major limitations in the proper management of schizophrenia are the non-adherence to the chronic regimen and the prolonged use of antipsychotics, often leading to metabolic and extrapyramidal side effects. In this context, long-active injectable (LAI) formulations for the sustained release of small drug doses represent an interesting approach. However, most available LAI for RSP, suffer of discontinuous drug release, with extensive initial lag phases, and rapid drug release, with the majority of the drug dose being delivered within 30 days or less. Indeed, conventional microspheres in the size range of 100 µm and larger show a typical cumulative RSP profile with an initially long lag phase (between 10 and 20 days) followed by an intermediate phase of sustained release (about 1 month) and a final phase of slow release of the residual amount of drug, which is typically insufficient for any in vivo activity [36]. As documented, this is also the case of the Risperdal Consta formulation [17, 18], which consequently requires the oral administration of RSP during the first 2–3-week post-microsphere injection to compensate for the lag phase. Several authors have tried to improve the release of RSP from polymeric microspheres by reducing their particle size. It was demonstrated that PLGA microspheres in the order of 10 µm in size have no lag phase but, given their small size, loose rapidly RSP so that the active release phase lasts 1–2 weeks [17, 18].

Differently, the release of RSP from both short and tall µPL follows a totally different kinetics. This is initially characterized by a relatively rapid release of the drug molecules mainly residing at the particles surface and is then followed by a prolonged and sustained release that is mainly diffusion driven. As expected, the increase in µPL thickness and polymer content for the tall over the short µPL reduced the initial burst effect and prolonged the release of RSP up to 100 days. Therefore, as opposed to microspheres, the tall µPL offers the unique advantage of providing the drug immediately from time zero (no lag phase) together with a sustained release for at least 3 months (long therapeutic action).

This unique release kinetic was the direct consequence of the exquisite control on µPL geometry and PLGA mass during the fabrication process. Differently from conventional microspheres, the µPL geometry can be finely tuned so to realize two µPL configurations: one is 10 µm high (short µPL), and the second one is 20 µm high (tall µPL). Both µPL have a square base with an edge length of 20 µm. On the contrary, polymeric microspheres are often characterized by a significant size range, as well as porous/rough surface which clearly impacts on their release characteristics [16–18, 36, 37].

The difference in thickness and polymer content between the two µPL configurations clearly affected the RSP loading, encapsulation efficiency, and release kinetics. Indeed, the significantly higher EE of the tall µPL has to be ascribed to their larger height along with the increased polymer content (20 µm, 60 mg), as opposed to the short µPL (10 µm, 20 mg). On the other hand, the comparable loading was expected, since LE is defined as the ratio between the weight of drug loaded into the µPL (higher for the tall µPL) and the total weight of the particles, which grows with the amount of PLGA. Even though the LE and EE are relatively low, these values are in line with what has been already published for risperidone-loaded microspheres of comparable size. Indeed, the literature shows that microspheres with a diameter of a few tens of microns present an EE of about 30% and a loading of a few points percentage [17, 18]. Only much larger microspheres, with an average diameter in the range of 100 μm and higher, have shown encapsulation efficiencies close to 90% and loading values in the range of 30–40% [16, 36, 37]. However, these larger particles require larger needle for the injection, and their higher loading does not necessarily translate in enhanced pharmacological performances, as described previously.

The µPL developed in this work, and in particular the tall particles, combine the benefits of the small PLGA microspheres (no lag phase) with those of the larger PLGA microspheres (prolonged release), thus extending the therapeutic window. This was clearly demonstrated by the blood concentration of RSP at 3-month post-administration, which was in the range of 4–6 ng/ml, with only 1.4 mg/kg initial administration. Other studies have demonstrated similar (or lower) plasma RSP concentrations at 3 months, but starting with an initial dose of 40 mg/kg [16]. Most importantly, the results of the behavioral studies demonstrated the efficacy of both short and tall µPL in improving the cognitive impairment characterizing Dys ± mice. Disruption of recency memory is a core symptom for patients affected by psychiatric disorders, including schizophrenia [38]. In rodents, this ability is assessed using the temporal order object recognition task (TOR), which evaluates the ability to discriminate two objects presented at two different moments [31, 32, 39]. Using the TOR tests, we previously demonstrated that chronic daily treatment with RSP (0.1 mg/kg) rescued cognitive impairments in Dys ± mice as similarly reported in human patients [20]. Within this context, the therapeutic efficacy and possible toxicity of one single injection of RSP-μPL was tested in Dys ± and their wild-type littermates (Dys + / +), subjected to TOR tests over a period of up to 3 months. By measuring the time spent to explore the two objects in the testing phase, the authors assessed if the treatment with RSP-μPL was able to improve the cognitive impairment characterizing Dys ± mice. Notably, treatments with short and tall µPL did not lead to any significant difference in the total time mice spent to explore the two objects during the 5-min test trial as documented by Figs. S4a, b, S5a, d, and S6a, b, suggesting that the µPL per se had no effect, thus giving important information on their safety profile. In addition, mice receiving empty µPL showed no discrimination, indicating the presence of a cognitive impairment and confirming that the particles per se have no effects. Notably, when µPL were administered to Dys ± mice, they had no negative outcome on the cognitive performances, reinforcing the fact that µPL did not lead to any side effect. A single i.p. deposition of short RSP-μPL was sufficient to rescue the cognitive deficit in Dys ± mice for up to 14 days, similarly to the conventional daily administration of 0.1 mg/kg free RSP. However, at 4-week post-deposition, the rescuing effect of the short RSP-μPL diminished, even if the trend was still visible. As documented by the biopharmaceutical analysis presented in Fig. 3, tall RSP-µPL can load larger amounts of drug and release it over longer periods. Therefore, the following natural step was that of testing in vivo therapeutic efficacy over longer times. A single injection of tall µPL was effective in rescuing the cognitive impairment of Dys ± mice up to 12 weeks, representing an advantage over the short RSP-µPL, where the TOR performances were improved only at 2-week post-injection. Indeed, this extended rescuing effect should be associated with the longer release of RSP from the tall over the short µPL. Importantly, a single injection of tall µPL not only helped restore the recency memory impairment, but led to a behavior of Dys ± mice similar to those of Dys + / + mice, thus confirming the efficacy of the treatment.

## Conclusions

In this study, square 20 × 20 μm PLGA μPL loaded with RSP were fabricated using a template-based fabrication approach. Specifically, two different μPL configurations were realized, characterized by a thickness of either 10 μm (short μPL) or 20 μm (tall μPL), and a polymer content of 20 mg and 60 mg, respectively. The difference in the height and polymer content of the μPL was reflected on the RSP encapsulation efficiency and release profile. Indeed, the higher PLGA mass and thickness of the tall μPL led to a larger drug encapsulation and longer sustained release as compared to short μPL. To test the in vivo efficacy, μPL were intraperitoneally injected in heterozygous knockout mice for dysbindin-1, a clinically relevant mouse model of cognitive and psychiatric liability, with proved evidence of modulatory responses to antipsychotic drugs in mice and human patients. At 2-, 4-, 8-, and 12-week post-treatment initiation, the ability of the animals to discriminate objects was assessed by performing temporal order object recognition tests. As for the in vitro pharmacological properties, the fine tuning of the μPL geometry and polymer content did affect the in vivo therapeutic performances. While short μPL were able to rescue the cognitive deficit of mice for up to 14 days, the tall μPL were effective up to 3 months. Finally, these findings suggest that RSP-μPL can be used as a long-acting depot capable of releasing antipsychotic drugs in a controlled manner up to several weeks and ameliorating the dysbindin-induced deficits with one single administration for at least 12 weeks. Further studies of µPL will investigate alternative methods of fabrication to improve LE and EE, without altering the particle efficacy. This would lead to the possibility of injecting even lower amounts of polymer, thus improving the overall safety of the µPL.

## Supplementary Information

Below is the link to the electronic supplementary material.

Supplementary file1 (DOCX 39477 KB)

## Data Availability

The raw data required to reproduce these results are available from the corresponding author upon reasonable request.
