# Supplementary material for: Long-lasting rescue of schizophrenia-relevant cognitive impairments via risperidone-loaded microPlates
Source: Drug Deliv Transl Res. 2022 Jan 1;12(8):1829–42. doi: 10.1007/s13346-021-01099-x (PMC9242964; doi:10.1007/s13346-021-01099-x)
Supplement: Supplementary file 1 — Supplementary file1 (DOCX 39477 KB) [file 13346_2021_1099_MOESM1_ESM.docx]

Supplementary Information

Long-lasting Rescue of Schizophrenia-relevant Cognitive Impairments via Risperidone-loaded microPlates

Drug Delivery and Translational Research

Special Issue: CRS Italia – Drug Delivery and Translational Research Activity

Elena Bellotti*, Gabriella Contarini, Federica Geraci, Sebastiano Alfio Torrisi, Cateno Piazza, Filippo Drago, Gian Marco Leggio, Francesco Papaleo, Paolo Decuzzi

*corresponding author: Laboratory of Nanotechnology for Precision Medicine, Istituto Italiano di Tecnologia, Via Morego 30, 16163 Genova, Italy. [elena.bellotti@iit.it](mailto:elena.bellotti@iit.it)

**Supplementary Materials and methods**

**Fabrication of the microspheres (µS)**

Risperidone-loaded microspheres (RSP-µS) were fabricated using a standard single emulsion procedure. Briefly, 20 mg of PLGA and 1 mg RSP was dissolved in 400 µl of chloroform. The polymer/dug mixture was homogenized in 6 ml of 2%PVA for 1 min at 10000 rpm (IKA® T10 Basic, ULTRA-TURRAX). The emulsion was then added to 8 ml 1% PVA solution and allowed to mix for 3 hours under magnetic stirring at 600 rpm to evaporate any remaining chloroform. The µS were then washed three times via centrifugation with deionized water and the final product stored at 4°C until further use.

**Morphological and dimensional characterization of the µS**

The morphology and dimensions of μS were characterized using different techniques. Specifically, μS shape and size analysis was carried out via scanning electron microcopy (SEM, JEOL JSM-6490LA). Briefly, a drop of sample was deposited on a silicon support and uniformly sputtered with gold to increase the contrast and reduce sample damaging. An acceleration voltage of 10 keV was used for SEM image acquisition. Additionally, μS average size and distribution of µS were obtained via dynamic light scattering (DLS) and volume impedance measurements. Briefly, µS were centrifuged at different speeds to obtain two separate populations of µS. µS with smaller dimensions were diluted using DI water and analyzed at 25°C using a Zetasizer Nano (Malvern, UK), ﻿equipped with a 4.5 mW laser diode and operating at 670 nm as a light source, and the scattered photons were detected at 173°. A third order cumulative fitting autocorrelation function is applied to measure the average size and size distributions. µS with bigger dimensions were analyzed using a Multisizer 4 COULTER particle counter (Beckman Coulter, CA). μS were resuspended in an electrolyte solution and analyzed using a 100 μm capillary.

**Biopharmaceutical characterization of the µS**

The RSP loading and encapsulation efficiency (LE and EE, respectively) into RSP-µS were evaluated by dissolving the μS in ACN/H_2_O (1:1 v/v) and analyzing the solution via high performance liquid chromatography (HPLC) (Agilent 1260 Infinity, Germany), equipped with a 100 μl loop. The mobile phase consisted in ACN + 0.1% TFA (v/v) and DI water + 0.1% TFA (v/v) at a ratio of 57/43 v/v and pumped at an isocratic flow rate of 0.3 ml/min. The analysis was performed by using a C18 column (2.1 x 10 mm, 3.5 μm particle size, Agilent Eclipse Plus, USA) at a detection wavelength of 280 nm. The amount of RSP loaded and encapsulated into the μPL was calculated interpolating a standard calibration curve. The loading (%LE) and encapsulation efficiency (%EE) were quantified using the following equations:

$\mathrm{LE}\left( \% \right)=\frac{RSP weight in particles}{Total weight of particles}\times100$ (S1)

$\mathrm{EE}\left( \% \right)=\frac{RSP weight in particles}{RSP initial feeding amount}\times100$ (S2)

To study RSP release, µS were incubated in 500 μl PBS under mechanical stirring at 37 °C. At predetermined time points, the μPL were centrifuged at 500 rpm, the supernatant removed and 500 μl of fresh PBS added. To quantify the amount of RSP released over time, the supernatant was analyzed via HPLC using the same method described above.

**Supplementary Results**

|  | ACN/DI water | PBS |
| --- | --- | --- |
| LoD | 0.53 ng/ml | 4.7 ng/ml |
| LoQ | 1.61 ng/ml | 14.1 ng/ml |

**Table S1** Limit of detection (LoQ) and limit of quantification (LoQ) of the HPLC method used to quantify RSP.


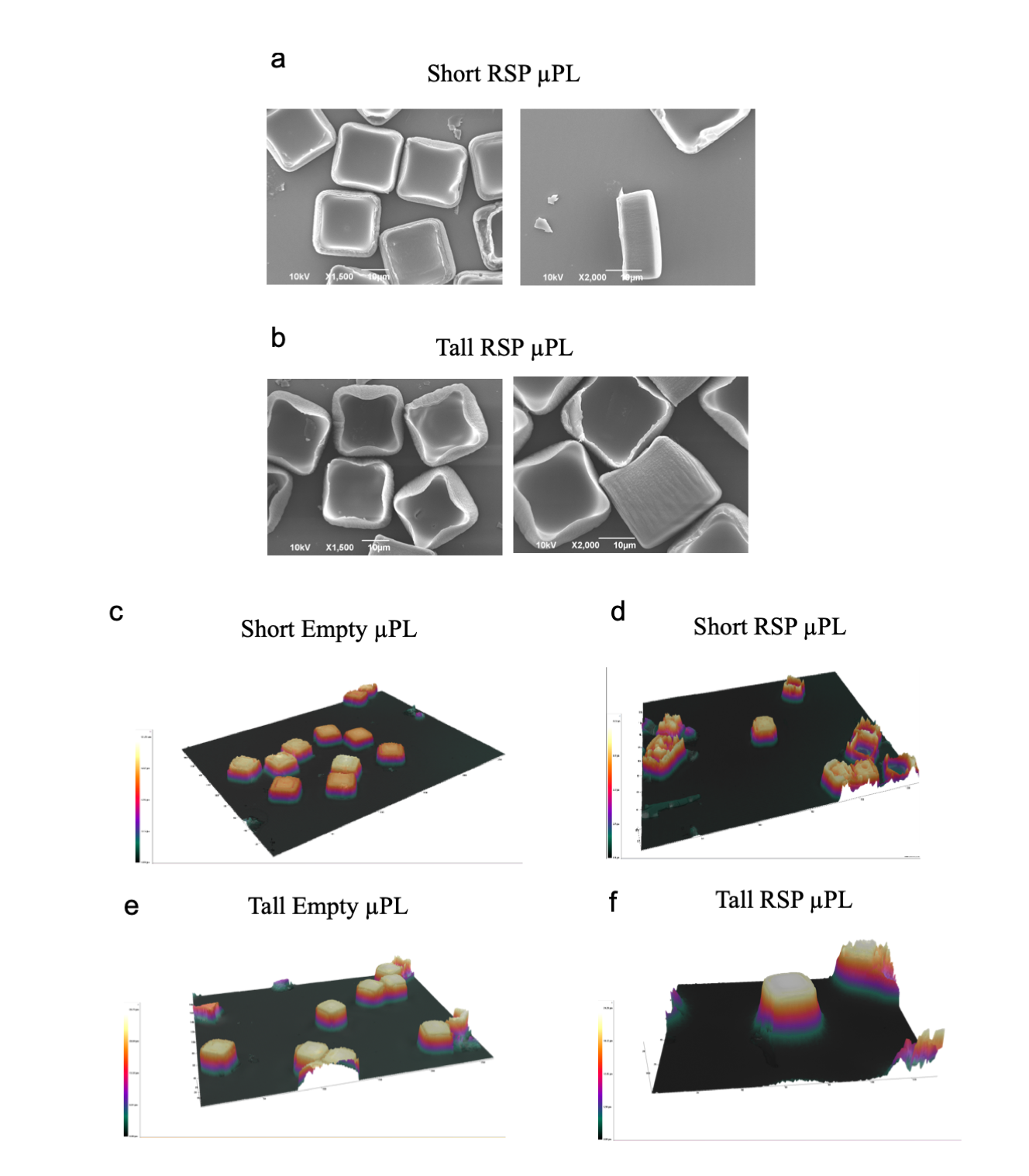


**Fig. S1** μPL morphological and dimensional characterization. (a) SEM micrographs of short and (b) tall RSP-μPL showing the characteristic dimensions of 20×20×10 and 20×20×20 μm, respectively. No differences between empty and RSP-μPL were highlighted in terms of shape and dimension. (c) False coloring three-dimensional reconstruction of short empty μPL, (d) short RSP-μPL, (e) tall empty μPL, and (f) tall RSP-μPL acquired by optical profilometer. The color intensity correlates with the local particle thickness


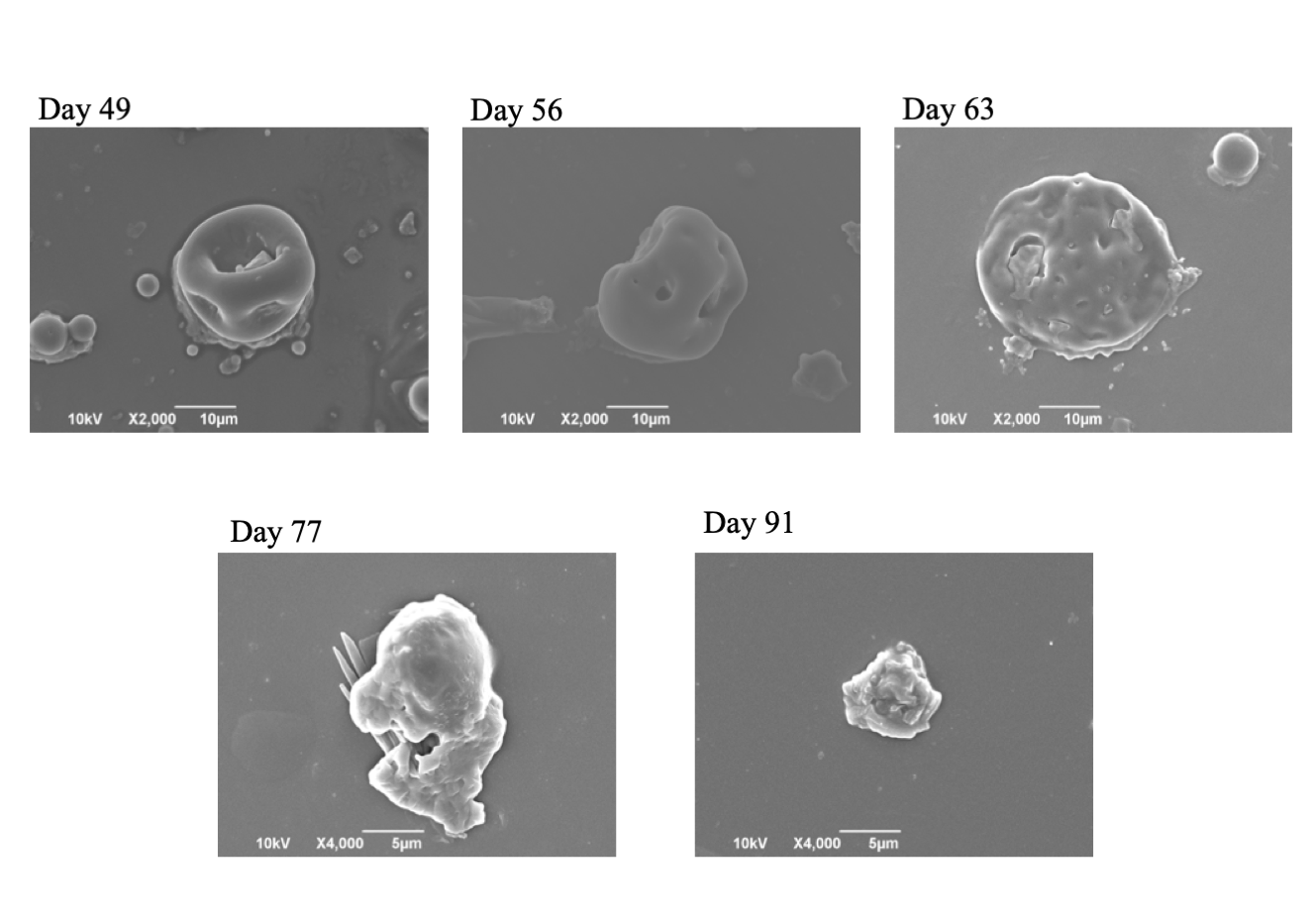


**Fig. S2** Degradation of tall μPL under physiological conditions. SEM images representing the degradation of tall μPL after 49, 56, 63, 77, and 91 days of incubation in PBS


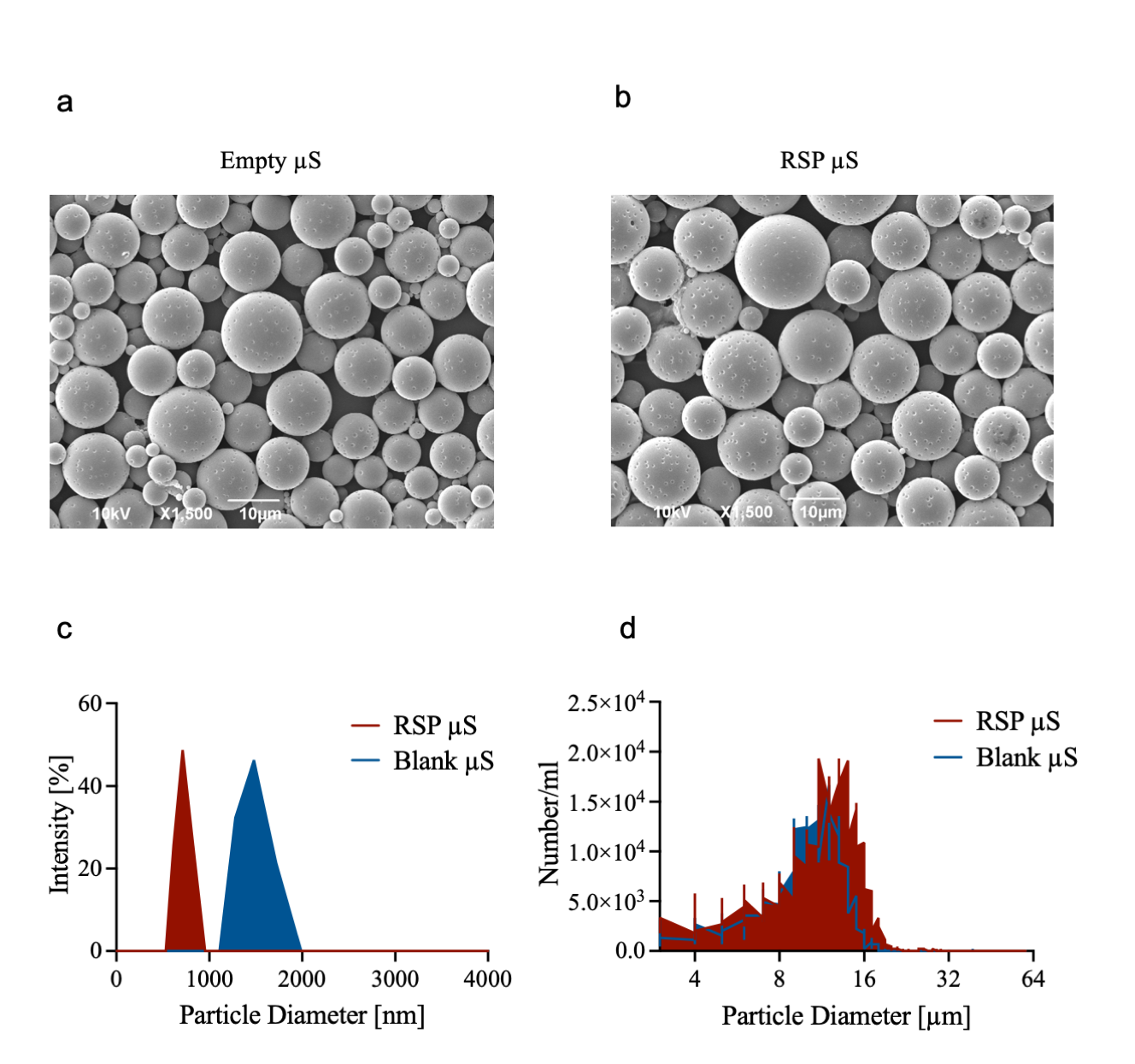


**Fig. S3** µS morphological and dimensional characterization. (a) SEM micrographs of empty and (b) RSP-µS showing spherical shape and porous surface of the particles characterized by two populations of dimensions. (c) DLS and (d) multisizer analysis highlighting the dimensions of the two populations of ~ 1-2 µm for the smaller µS and ~ 10 µm for the bigger µS


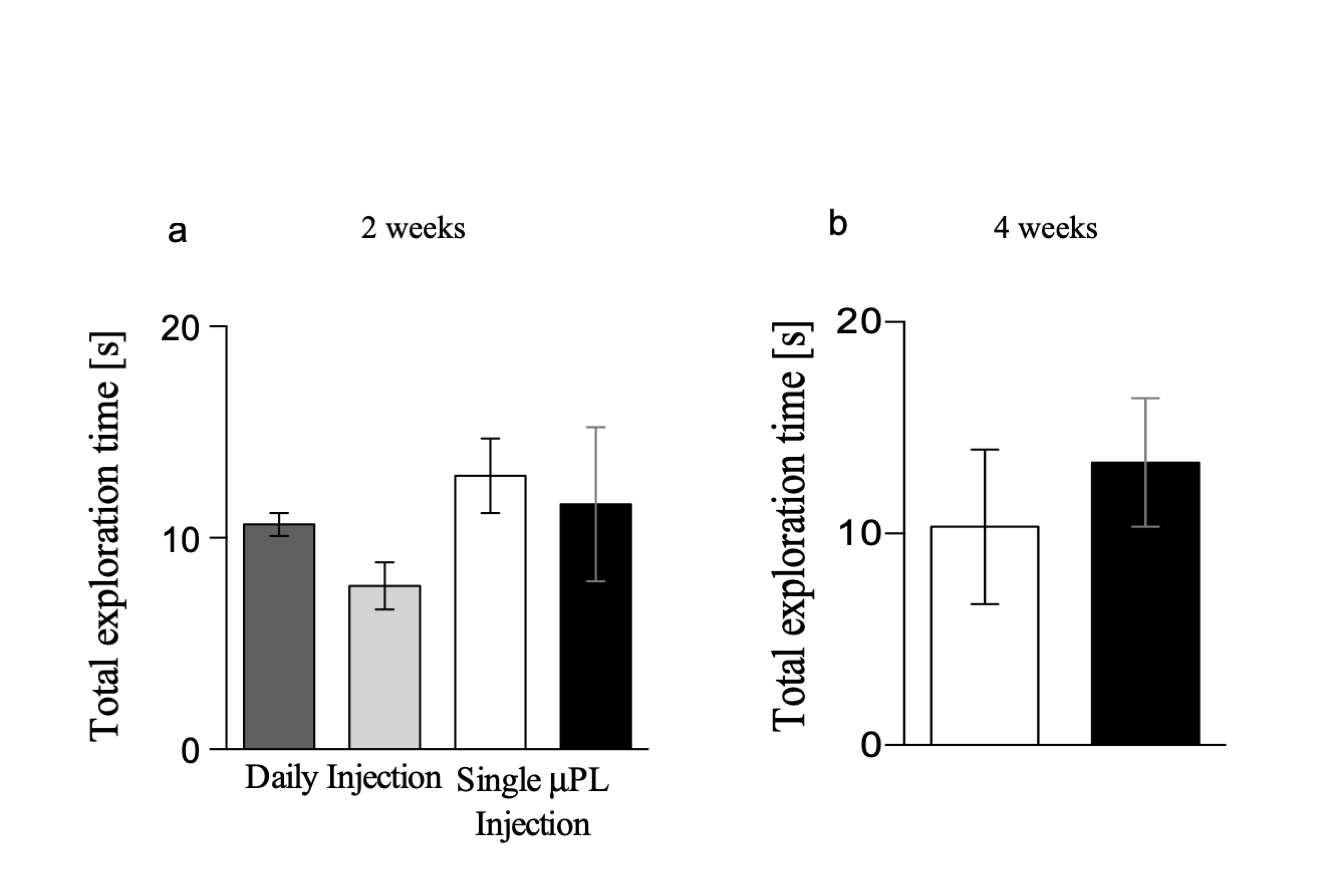


**Fig. S4** Total exploration time. Short empty or RSP-μPL did not affect the total amount mice spent exploring the two objects presented during the 5-min test trial at (a) 2, and (b) 4 weeks post injection (Free RSP: Dys +/- n=7, vehicle: Dys +/- n=7, *empty μPLs*: Dys +/- n=6, *RSP-μPL:* Dys+/- n=6)


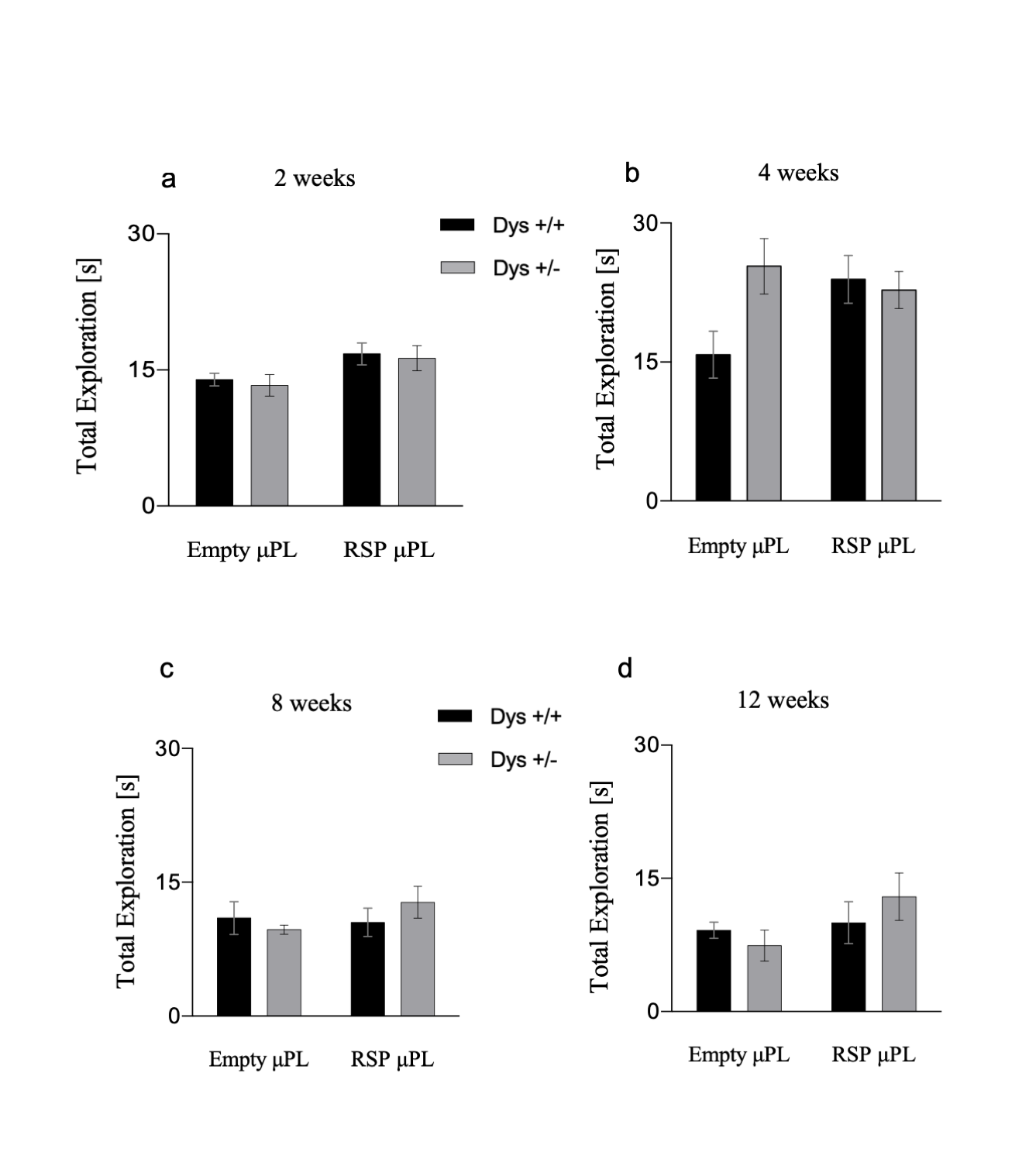


**Fig. S5** Total exploration time. Tall empty or RSP-μPL did not affect the total amount mice spent exploring the two objects presented during the 5-min test trial at (a) 2, (b) 4, (c) 8, and (d) 12 weeks post injection (*empty μPLs*: Dys +/+ n=6, Dys+/- n=6; *RSP-μPLs:* Dys+/+ n=6, Dys +/- n=6)


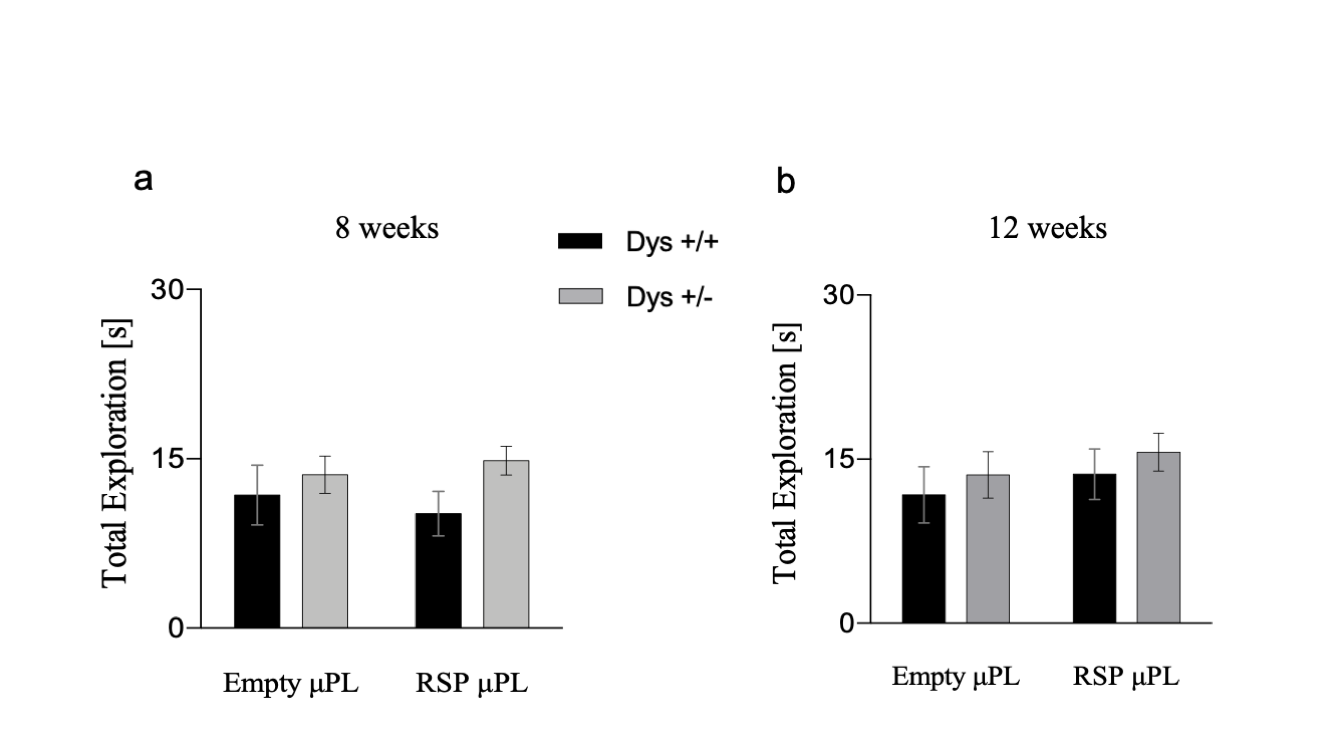


**Fig. S6** Total exploration time**.** Tall empty or RSP-μPL did not affect the total time mice spent exploring the two objects presented during the 5-min test trial at (a) 8 and (b) 12 weeks post injection (*empty μPLs*: Dys +/+ n=6, Dys +/- n=10; *RSP-μPLs:*  Dys +/+ n=6, Dys +/- n=10)


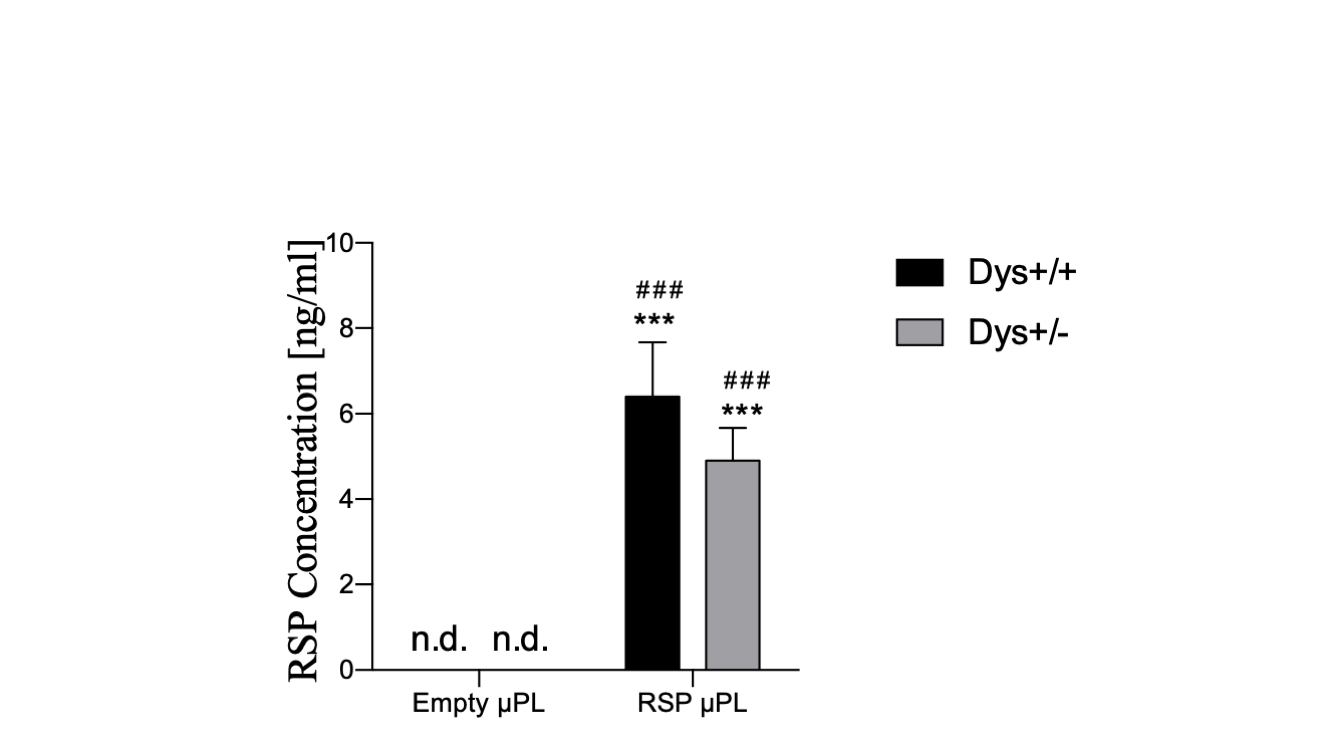


**Fig. S7** *In vivo* RSP concentration. Serum concentrations of RSP in Dys +/+ and Dys +/- evaluated at 12 weeks post injection of tall RSP-μPL. Bonferroni post-hoc: ***p<0.0001 vs empty μPL Dys +/+; ### p<0.0001 vs empty μPL Dys +/-

**Supplementary statistical analysis**

The statistical analysis (one-way ANOVA) is expressed as: p values <0.05 (*), <0.001 (***), < 0.0001 (§), and <0.00001 (#). “ns” stands for not statistically significant.

|  | Short µPL vs Tall µPL | | Short µPL vs µS | µS vs Tall µPL |
| --- | --- | --- | --- | --- |
| 1 day | | *** | * | *** |
| 40 days | | # | ns | § |
| 50 days | | # | - | - |

**Table S2** In vitro RSP release from short µPL, tall µPL, and µS
